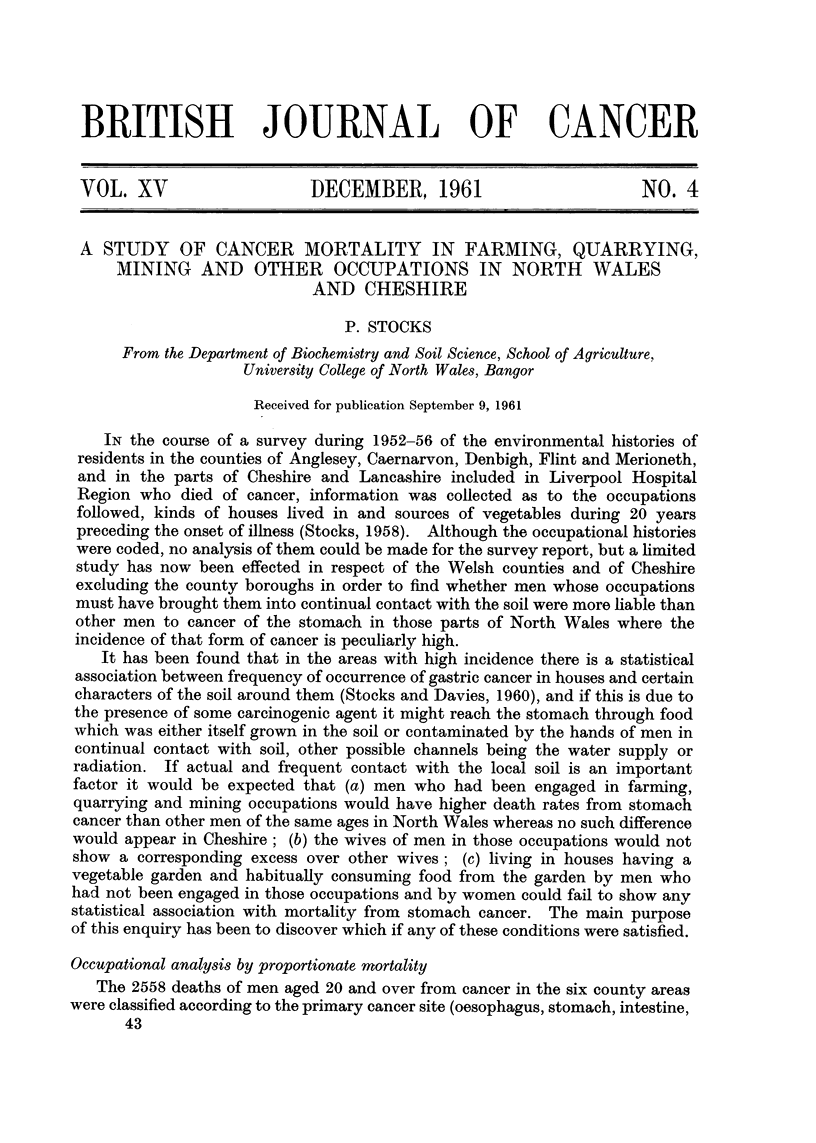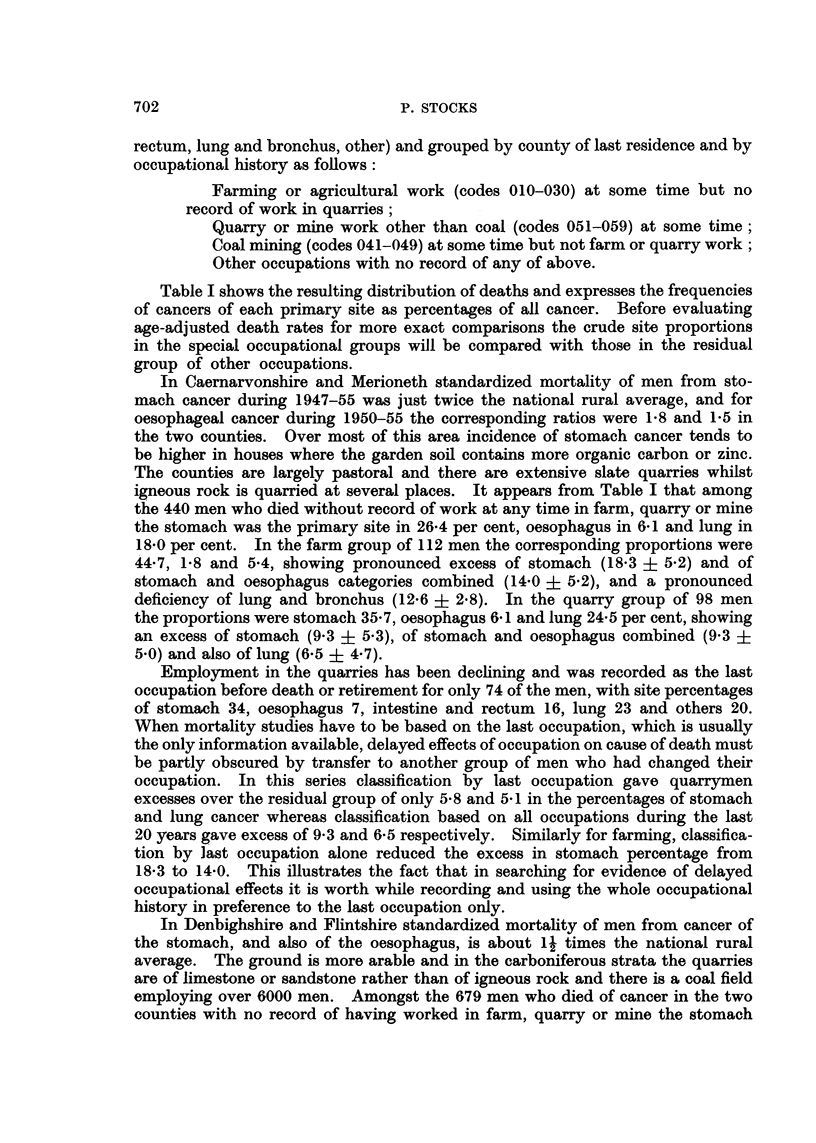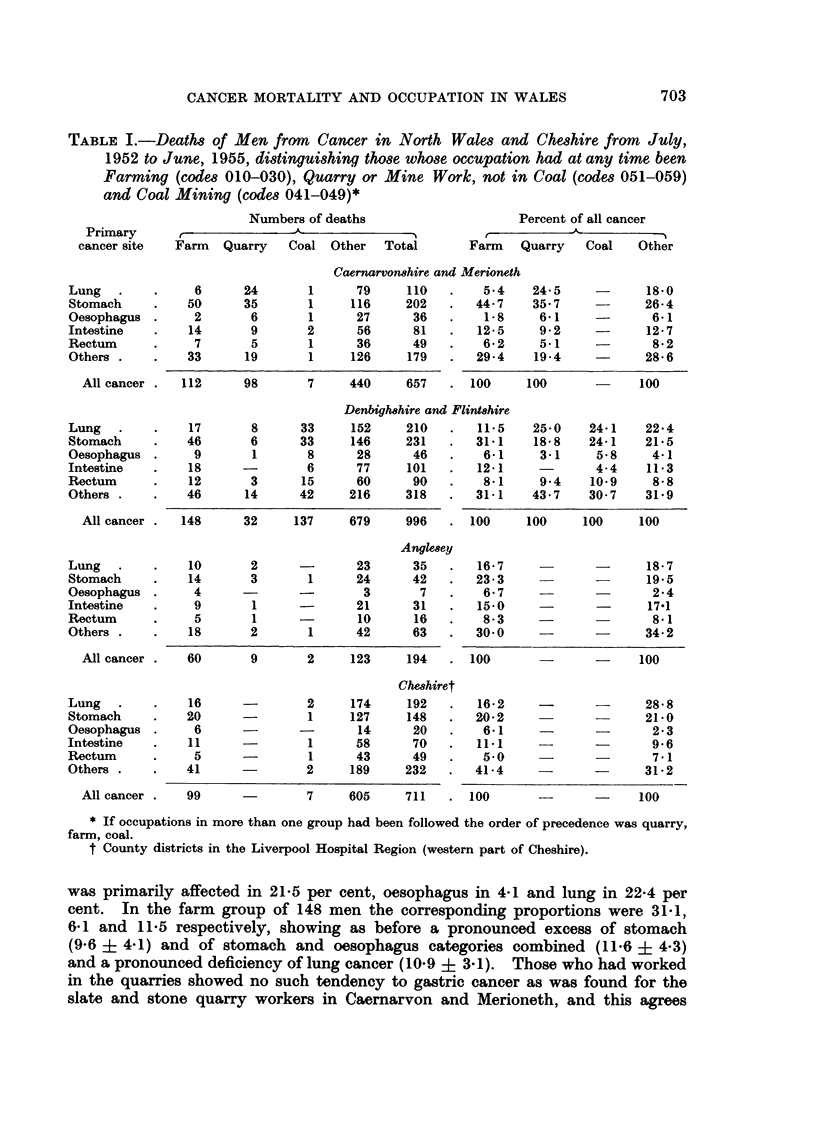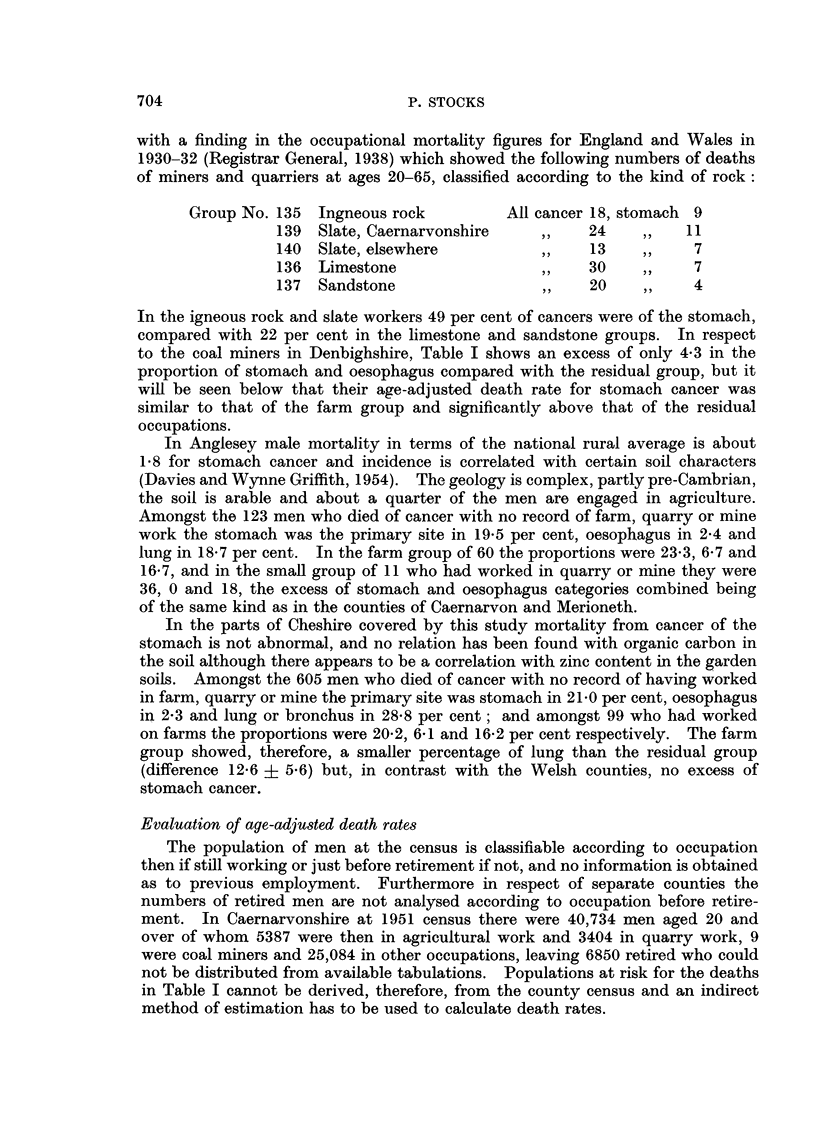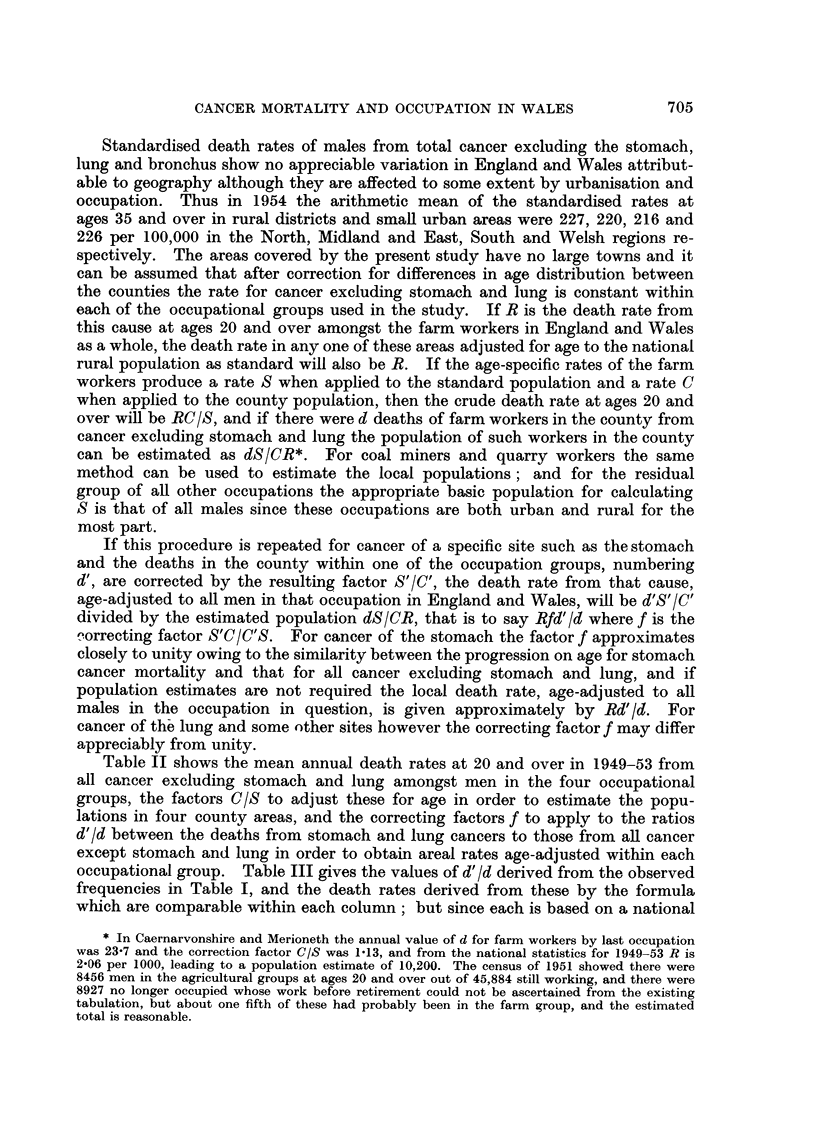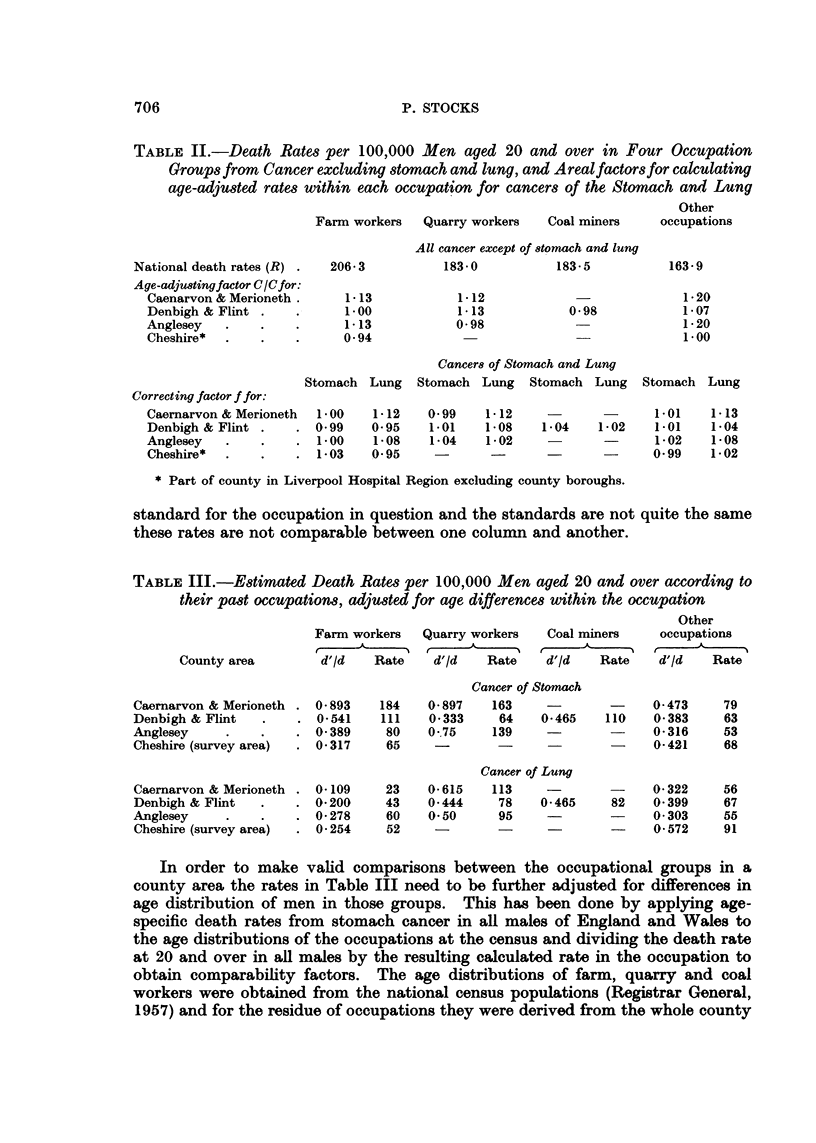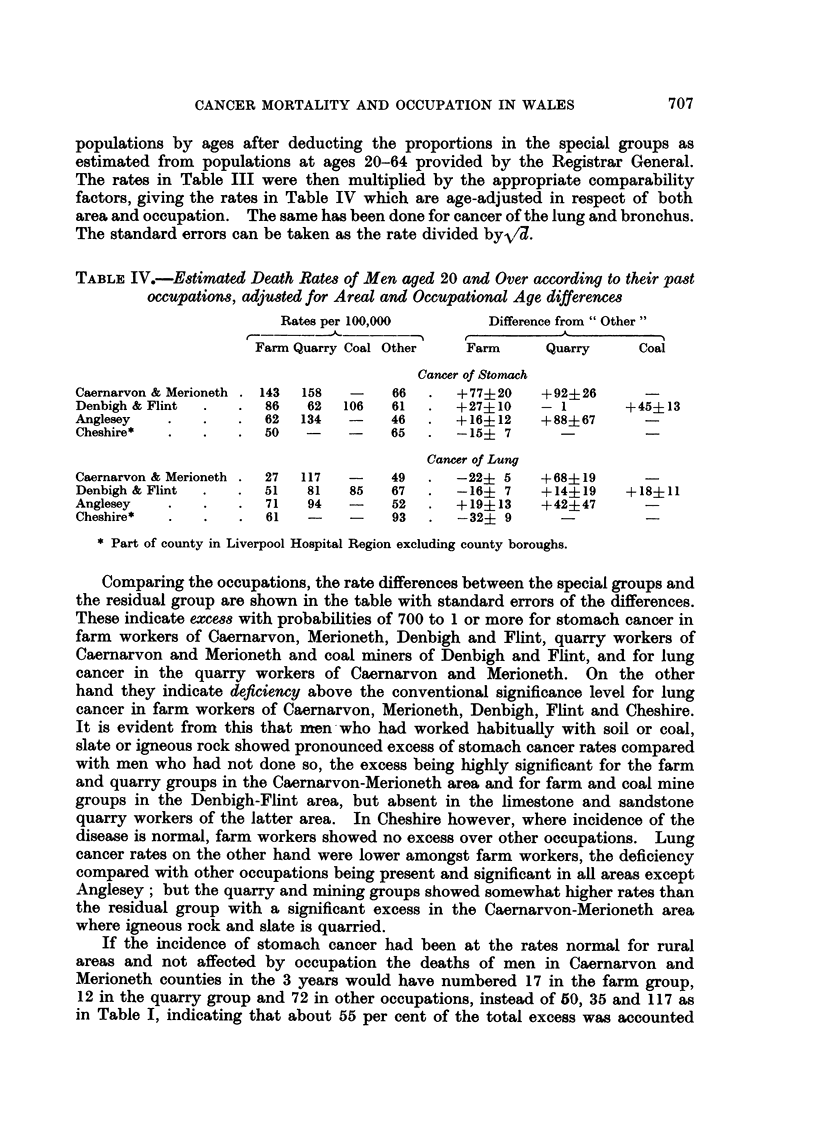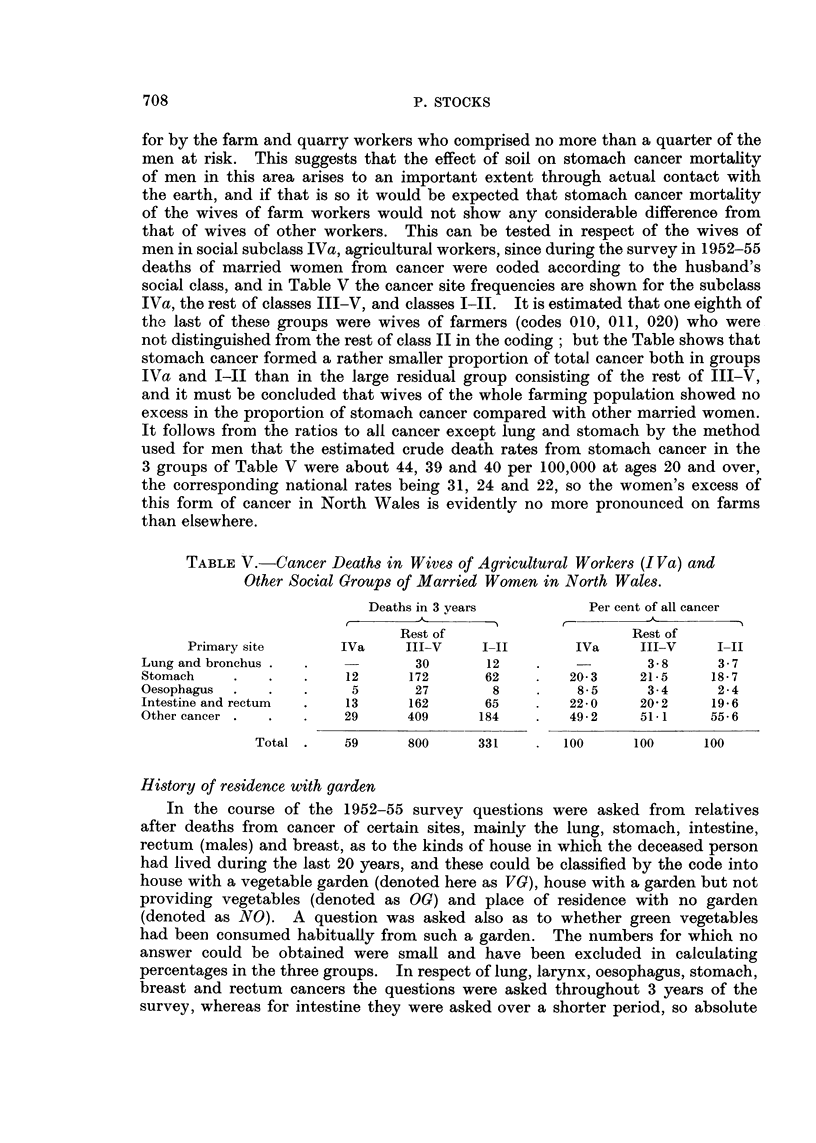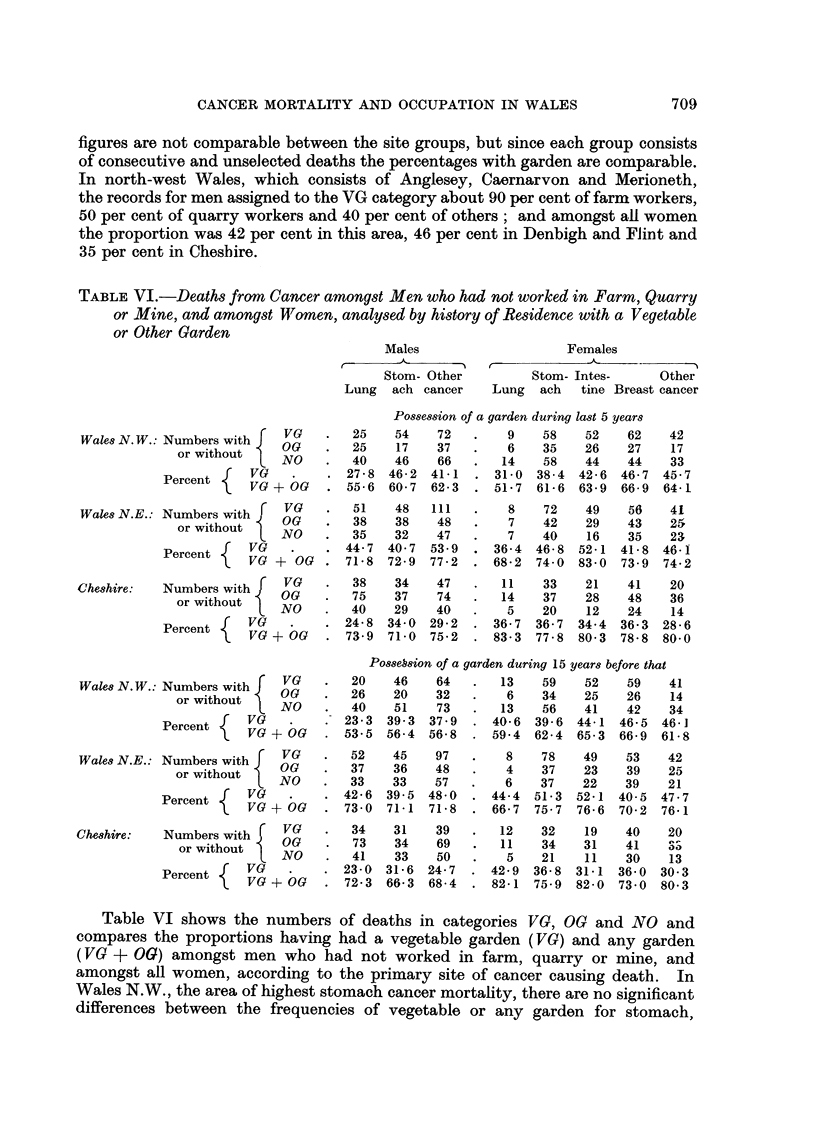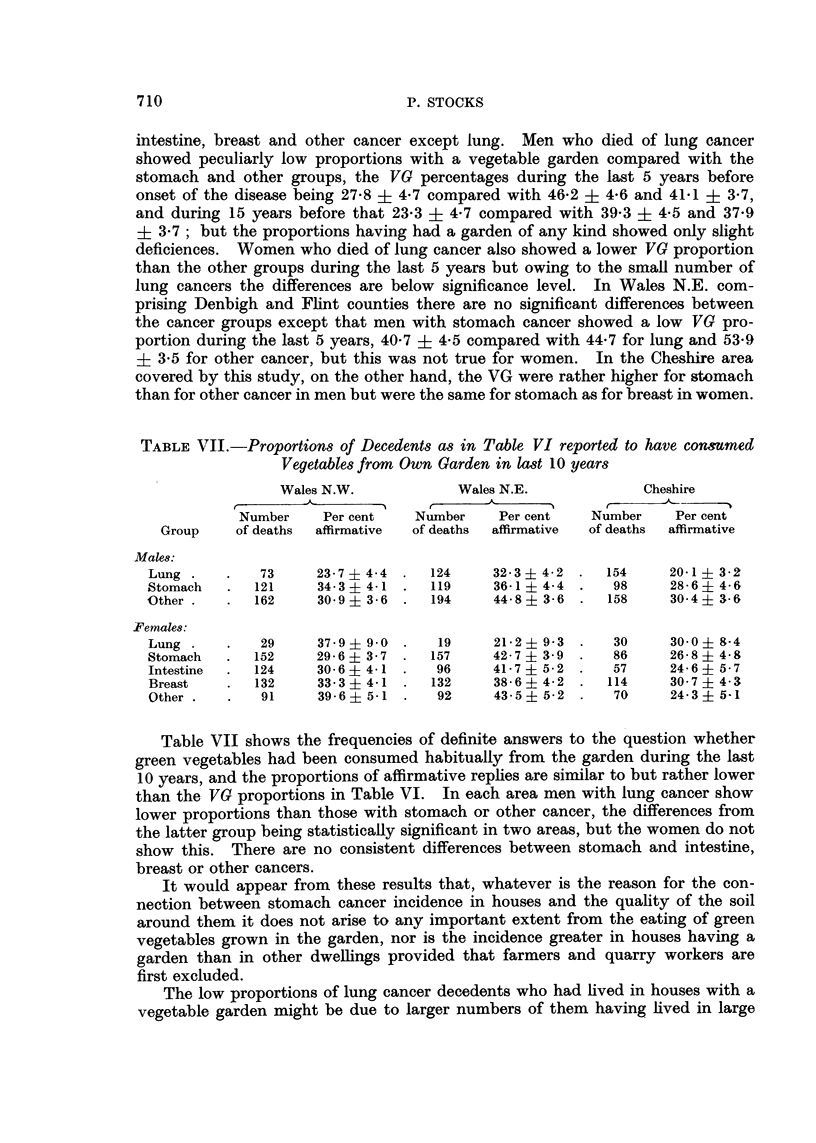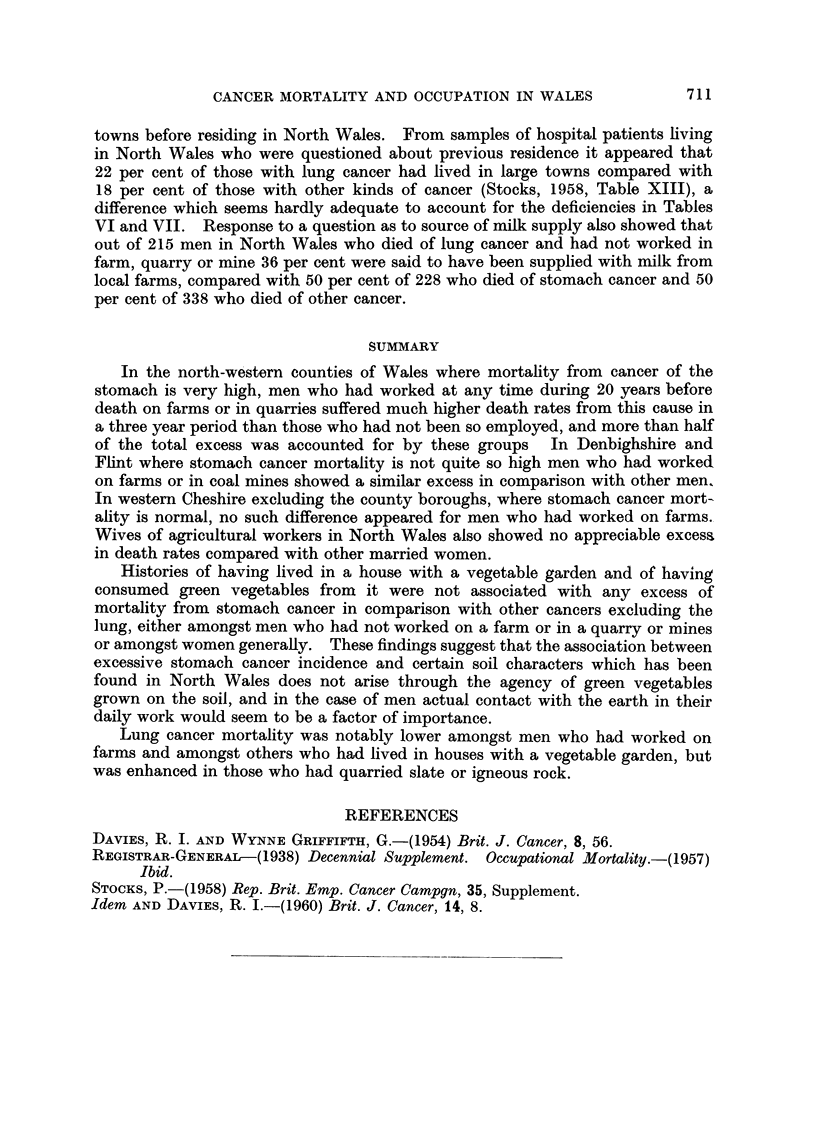# A Study of Cancer Mortality in Farming, Quarrying, Mining and other Occupations in North Wales and Cheshire

**DOI:** 10.1038/bjc.1961.80

**Published:** 1961-12

**Authors:** P. Stocks


### BRITISH JOURNAL OF CANCER

VOL. XV DECEMBER, 1961 NO. 4

### A STUDY OF CANCER MORTALITY IN FARMING, QUARRYING, MINING AND OTHER OCCUPATIONS IN NORTH WALES AND CHESHIRE

P. STOCKS

*From the Department of Biochemistry and Soil Science, School of Agriculture, University College of North Wales, Bangor*

Received for publication September 9, 1961

In the course of a survey during 1952–56 of the environmental histories of residents in the counties of Anglesey, Caernarvon, Denbigh, Flint and Merioneth, and in the parts of Cheshire and Lancashire included in Liverpool Hospital Region who died of cancer, information was collected as to the occupations followed, kinds of houses lived in and sources of vegetables during 20 years preceding the onset of illness (Stocks, 1958). Although the occupational histories were coded, no analysis of them could be made for the survey report, but a limited study has now been effected in respect of the Welsh counties and of Cheshire excluding the county boroughs in order to find whether men whose occupations must have brought them into continual contact with the soil were more liable than other men to cancer of the stomach in those parts of North Wales where the incidence of that form of cancer is peculiarly high.

It has been found that in the areas with high incidence there is a statistical association between frequency of occurrence of gastric cancer in houses and certain characters of the soil around them (Stocks and Davies, 1960), and if this is due to the presence of some carcinogenic agent it might reach the stomach through food which was either itself grown in the soil or contaminated by the hands of men in continual contact with soil, other possible channels being the water supply or radiation. If actual and frequent contact with the local soil is an important factor it would be expected that (*a*) men who had been engaged in farming, quarrying and mining occupations would have higher death rates from stomach cancer than other men of the same ages in North Wales whereas no such difference would appear in Cheshire ; (*b*) the wives of men in those occupations would not show a corresponding excess over other wives ; (*c*) living in houses having a vegetable garden and habitually consuming food from the garden by men who had not been engaged in those occupations and by women could fail to show any statistical association with mortality from stomach cancer. The main purpose of this enquiry has been to discover which if any of these conditions were satisfied.

### *Occupational analysis by proportionate mortality*

The 2558 deaths of men aged 20 and over from cancer in the six county areas were classified according to the primary cancer site (oesophagus, stomach, intestine, rectum, lung and bronchus, other) and grouped by county of last residence and by occupational history as follows :

Farming or agricultural work (codes 010–030) at some time but no record of work in quarries ;
Quarry or mine work other than coal (codes 051–059) at some time ;
Coal mining (codes 041–049) at some time but not farm or quarry work ;
Other occupations with no record of any of above.

Table I shows the resulting distribution of deaths and expresses the frequencies of cancers of each primary site as percentages of all cancer. Before evaluating age-adjusted death rates for more exact comparisons the crude site proportions in the special occupational groups will be compared with those in the residual group of other occupations.

In Caernarvonshire and Merioneth standardized mortality of men from stomach cancer during 1947–55 was just twice the national rural average, and for oesophageal cancer during 1950–55 the corresponding ratios were 1·8 and 1·5 in the two counties. Over most of this area incidence of stomach cancer tends to be higher in houses where the garden soil contains more organic carbon or zinc. The counties are largely pastoral and there are extensive slate quarries whilst igneous rock is quarried at several places. It appears from Table I that among the 440 men who died without record of work at any time in farm, quarry or mine the stomach was the primary site in 26·4 per cent, oesophagus in 6·1 and lung in 18·0 per cent. In the farm group of 112 men the corresponding proportions were 44·7, 1·8 and 5·4, showing pronounced excess of stomach (18·3 $\pm$ 5·2) and of stomach and oesophagus categories combined (14·0 $\pm$ 5·2), and a pronounced deficiency of lung and bronchus (12·6 $\pm$ 2·8). In the quarry group of 98 men the proportions were stomach 35·7, oesophagus 6·1 and lung 24·5 per cent, showing an excess of stomach (9·3 $\pm$ 5·3), of stomach and oesophagus combined (9·3 $\pm$ 5·0) and also of lung (6·5 $\pm$ 4·7).

Employment in the quarries has been declining and was recorded as the last occupation before death or retirement for only 74 of the men, with site percentages of stomach 34, oesophagus 7, intestine and rectum 16, lung 23 and others 20. When mortality studies have to be based on the last occupation, which is usually the only information available, delayed effects of occupation on cause of death must be partly obscured by transfer to another group of men who had changed their occupation. In this series classification by last occupation gave quarrymen excesses over the residual group of only 5·8 and 5·1 in the percentages of stomach and lung cancer whereas classification based on all occupations during the last 20 years gave excess of 9·3 and 6·5 respectively. Similarly for farming, classification by last occupation alone reduced the excess in stomach percentage from 18·3 to 14·0. This illustrates the fact that in searching for evidence of delayed occupational effects it is worth while recording and using the whole occupational history in preference to the last occupation only.

In Denbighshire and Flintshire standardized mortality of men from cancer of the stomach, and also of the oesophagus, is about 1½ times the national rural average. The ground is more arable and in the carboniferous strata the quarries are of limestone or sandstone rather than of igneous rock and there is a coal field employing over 6000 men. Amongst the 679 men who died of cancer in the two counties with no record of having worked in farm, quarry or mine the stomach

TABLE I.—*Deaths of Men from Cancer in North Wales and Cheshire from July,* 1952 *to June,* 1955, *distinguishing those whose occupation had at any time been Farming* (*codes* 010–030), *Quarry or Mine Work, not in Coal* (*codes* 051–059) *and Coal Mining* (*codes* 041–049)*

| Primary cancer site | Numbers of deaths | | | | | Percent of all cancer | | | |
|---|---|---|---|---|---|---|---|---|---|
| | Farm | Quarry | Coal | Other | Total | Farm | Quarry | Coal | Other |
| | | | | *Caernarvonshire and Merioneth* | | | | | |
| Lung | 6 | 24 | 1 | 79 | 110 | 5·4 | 24·5 | — | 18·0 |
| Stomach | 50 | 35 | 1 | 116 | 202 | 44·7 | 35·7 | — | 26·4 |
| Oesophagus | 2 | 6 | 1 | 27 | 36 | 1·8 | 6·1 | — | 6·1 |
| Intestine | 14 | 9 | 2 | 56 | 81 | 12·5 | 9·2 | — | 12·7 |
| Rectum | 7 | 5 | 1 | 36 | 49 | 6·2 | 5·1 | — | 8·2 |
| Others | 33 | 19 | 1 | 126 | 179 | 29·4 | 19·4 | — | 28·6 |
| All cancer | 112 | 98 | 7 | 440 | 657 | 100 | 100 | — | 100 |
| | | | | *Denbighshire and Flintshire* | | | | | |
| Lung | 17 | 8 | 33 | 152 | 210 | 11·5 | 25·0 | 24·1 | 22·4 |
| Stomach | 46 | 6 | 33 | 146 | 231 | 31·1 | 18·8 | 24·1 | 21·5 |
| Oesophagus | 9 | 1 | 8 | 28 | 46 | 6·1 | 3·1 | 5·8 | 4·1 |
| Intestine | 18 | — | 6 | 77 | 101 | 12·1 | — | 4·4 | 11·3 |
| Rectum | 12 | 3 | 15 | 60 | 90 | 8·1 | 9·4 | 10·9 | 8·8 |
| Others | 46 | 14 | 42 | 216 | 318 | 31·1 | 43·7 | 30·7 | 31·9 |
| All cancer | 148 | 32 | 137 | 679 | 996 | 100 | 100 | 100 | 100 |
| | | | | | *Anglesey* | | | | |
| Lung | 10 | 2 | — | 23 | 35 | 16·7 | — | — | 18·7 |
| Stomach | 14 | 3 | 1 | 24 | 42 | 23·3 | — | — | 19·5 |
| Oesophagus | 4 | — | — | 3 | 7 | 6·7 | — | — | 2·4 |
| Intestine | 9 | 1 | — | 21 | 31 | 15·0 | — | — | 17·1 |
| Rectum | 5 | 1 | — | 10 | 16 | 8·3 | — | — | 8·1 |
| Others | 18 | 2 | 1 | 42 | 63 | 30·0 | — | — | 34·2 |
| All cancer | 60 | 9 | 2 | 123 | 194 | 100 | — | — | 100 |
| | | | | | *Cheshire*† | | | | |
| Lung | 16 | — | 2 | 174 | 192 | 16·2 | — | — | 28·8 |
| Stomach | 20 | — | 1 | 127 | 148 | 20·2 | — | — | 21·0 |
| Oesophagus | 6 | — | — | 14 | 20 | 6·1 | — | — | 2·3 |
| Intestine | 11 | — | 1 | 58 | 70 | 11·1 | — | — | 9·6 |
| Rectum | 5 | — | 1 | 43 | 49 | 5·0 | — | — | 7·1 |
| Others | 41 | — | 2 | 189 | 232 | 41·4 | — | — | 31·2 |
| All cancer | 99 | — | 7 | 605 | 711 | 100 | — | — | 100 |

* If occupations in more than one group had been followed the order of precedence was quarry, farm, coal.

† County districts in the Liverpool Hospital Region (western part of Cheshire).

was primarily affected in 21·5 per cent, oesophagus in 4·1 and lung in 22·4 per cent. In the farm group of 148 men the corresponding proportions were 31·1, 6·1 and 11·5 respectively, showing as before a pronounced excess of stomach (9·6 $\pm$ 4·1) and of stomach and oesophagus categories combined (11·6 $\pm$ 4·3) and a pronounced deficiency of lung cancer (10·9 $\pm$ 3·1). Those who had worked in the quarries showed no such tendency to gastric cancer as was found for the slate and stone quarry workers in Caernarvon and Merioneth, and this agrees with a finding in the occupational mortality figures for England and Wales in 1930–32 (Registrar General, 1938) which showed the following numbers of deaths of miners and quarriers at ages 20–65, classified according to the kind of rock :

| | | | | | | |
|---|---|---|---|---|---|---|
| Group No. 135 | Ingneous rock | All cancer | 18, | stomach | 9 |
| 139 | Slate, Caernarvonshire | ,, | 24 | ,, | 11 |
| 140 | Slate, elsewhere | ,, | 13 | ,, | 7 |
| 136 | Limestone | ,, | 30 | ,, | 7 |
| 137 | Sandstone | ,, | 20 | ,, | 4 |

In the igneous rock and slate workers 49 per cent of cancers were of the stomach, compared with 22 per cent in the limestone and sandstone groups. In respect to the coal miners in Denbighshire, Table I shows an excess of only 4·3 in the proportion of stomach and oesophagus compared with the residual group, but it will be seen below that their age-adjusted death rate for stomach cancer was similar to that of the farm group and significantly above that of the residual occupations.

In Anglesey male mortality in terms of the national rural average is about 1·8 for stomach cancer and incidence is correlated with certain soil characters (Davies and Wynne Griffith, 1954). The geology is complex, partly pre-Cambrian, the soil is arable and about a quarter of the men are engaged in agriculture. Amongst the 123 men who died of cancer with no record of farm, quarry or mine work the stomach was the primary site in 19·5 per cent, oesophagus in 2·4 and lung in 18·7 per cent. In the farm group of 60 the proportions were 23·3, 6·7 and 16·7, and in the small group of 11 who had worked in quarry or mine they were 36, 0 and 18, the excess of stomach and oesophagus categories combined being of the same kind as in the counties of Caernarvon and Merioneth.

In the parts of Cheshire covered by this study mortality from cancer of the stomach is not abnormal, and no relation has been found with organic carbon in the soil although there appears to be a correlation with zinc content in the garden soils. Amongst the 605 men who died of cancer with no record of having worked in farm, quarry or mine the primary site was stomach in 21·0 per cent, oesophagus in 2·3 and lung or bronchus in 28·8 per cent ; and amongst 99 who had worked on farms the proportions were 20·2, 6·1 and 16·2 per cent respectively. The farm group showed, therefore, a smaller percentage of lung than the residual group (difference 12·6 $\pm$ 5·6) but, in contrast with the Welsh counties, no excess of stomach cancer.

*Evaluation of age-adjusted death rates*

The population of men at the census is classifiable according to occupation then if still working or just before retirement if not, and no information is obtained as to previous employment. Furthermore in respect of separate counties the numbers of retired men are not analysed according to occupation before retirement. In Caernarvonshire at 1951 census there were 40,734 men aged 20 and over of whom 5387 were then in agricultural work and 3404 in quarry work, 9 were coal miners and 25,084 in other occupations, leaving 6850 retired who could not be distributed from available tabulations. Populations at risk for the deaths in Table I cannot be derived, therefore, from the county census and an indirect method of estimation has to be used to calculate death rates.

Standardised death rates of males from total cancer excluding the stomach, lung and bronchus show no appreciable variation in England and Wales attributable to geography although they are affected to some extent by urbanisation and occupation. Thus in 1954 the arithmetic mean of the standardised rates at ages 35 and over in rural districts and small urban areas were 227, 220, 216 and 226 per 100,000 in the North, Midland and East, South and Welsh regions respectively. The areas covered by the present study have no large towns and it can be assumed that after correction for differences in age distribution between the counties the rate for cancer excluding stomach and lung is constant within each of the occupational groups used in the study. If $R$ is the death rate from this cause at ages 20 and over amongst the farm workers in England and Wales as a whole, the death rate in any one of these areas adjusted for age to the national rural population as standard will also be $R$. If the age-specific rates of the farm workers produce a rate $S$ when applied to the standard population and a rate $C$ when applied to the county population, then the crude death rate at ages 20 and over will be $RC/S$, and if there were $d$ deaths of farm workers in the county from cancer excluding stomach and lung the population of such workers in the county can be estimated as $dS/CR$*. For coal miners and quarry workers the same method can be used to estimate the local populations ; and for the residual group of all other occupations the appropriate basic population for calculating $S$ is that of all males since these occupations are both urban and rural for the most part.

If this procedure is repeated for cancer of a specific site such as the stomach and the deaths in the county within one of the occupation groups, numbering $d'$, are corrected by the resulting factor $S'/C'$, the death rate from that cause, age-adjusted to all men in that occupation in England and Wales, will be $d'S'/C'$ divided by the estimated population $dS/CR$, that is to say $Rfd'/d$ where $f$ is the correcting factor $S'C/C'S$. For cancer of the stomach the factor $f$ approximates closely to unity owing to the similarity between the progression on age for stomach cancer mortality and that for all cancer excluding stomach and lung, and if population estimates are not required the local death rate, age-adjusted to all males in the occupation in question, is given approximately by $Rd'/d$. For cancer of the lung and some other sites however the correcting factor $f$ may differ appreciably from unity.

Table II shows the mean annual death rates at 20 and over in 1949–53 from all cancer excluding stomach and lung amongst men in the four occupational groups, the factors $C/S$ to adjust these for age in order to estimate the populations in four county areas, and the correcting factors $f$ to apply to the ratios $d'/d$ between the deaths from stomach and lung cancers to those from all cancer except stomach and lung in order to obtain areal rates age-adjusted within each occupational group. Table III gives the values of $d'/d$ derived from the observed frequencies in Table I, and the death rates derived from these by the formula which are comparable within each column ; but since each is based on a national

* In Caernarvonshire and Merioneth the annual value of $d$ for farm workers by last occupation was 23·7 and the correction factor $C/S$ was 1·13, and from the national statistics for 1949–53 $R$ is 2·06 per 1000, leading to a population estimate of 10,200. The census of 1951 showed there were 8456 men in the agricultural groups at ages 20 and over out of 45,884 still working, and there were 8927 no longer occupied whose work before retirement could not be ascertained from the existing tabulation, but about one fifth of these had probably been in the farm group, and the estimated total is reasonable.

TABLE II.—*Death Rates per 100,000 Men aged 20 and over in Four Occupation Groups from Cancer excluding stomach and lung, and Areal factors for calculating age-adjusted rates within each occupation for cancers of the Stomach and Lung*

| | Farm workers | Quarry workers | Coal miners | Other occupations |
|---|---|---|---|---|
| | | *All cancer except of stomach and lung* | | |
| National death rates (*R*) | 206·3 | 183·0 | 183·5 | 163·9 |
| *Age-adjusting factor C/C for:* | | | | |
| Caenarvon & Merioneth | 1·13 | 1·12 | — | 1·20 |
| Denbigh & Flint | 1·00 | 1·13 | 0·98 | 1·07 |
| Anglesey | 1·13 | 0·98 | — | 1·20 |
| Cheshire* | 0·94 | — | — | 1·00 |

| | Farm workers | | Quarry workers | | Coal miners | | Other occupations | |
|---|---|---|---|---|---|---|---|---|
| | | | *Cancers of Stomach and Lung* | | | | | |
| | Stomach | Lung | Stomach | Lung | Stomach | Lung | Stomach | Lung |
| *Correcting factor f for:* | | | | | | | | |
| Caernarvon & Merioneth | 1·00 | 1·12 | 0·99 | 1·12 | — | — | 1·01 | 1·13 |
| Denbigh & Flint | 0·99 | 0·95 | 1·01 | 1·08 | 1·04 | 1·02 | 1·01 | 1·04 |
| Anglesey | 1·00 | 1·08 | 1·04 | 1·02 | — | — | 1·02 | 1·08 |
| Cheshire* | 1·03 | 0·95 | — | — | — | — | 0·99 | 1·02 |

* Part of county in Liverpool Hospital Region excluding county boroughs.

standard for the occupation in question and the standards are not quite the same these rates are not comparable between one column and another.

TABLE III.—*Estimated Death Rates per 100,000 Men aged 20 and over according to their past occupations, adjusted for age differences within the occupation*

| County area | Farm workers *d'/d* | Farm workers Rate | Quarry workers *d'/d* | Quarry workers Rate | Coal miners *d'/d* | Coal miners Rate | Other occupations *d'/d* | Other occupations Rate |
|---|---|---|---|---|---|---|---|---|
| | | | | *Cancer of Stomach* | | | | |
| Caernarvon & Merioneth | 0·893 | 184 | 0·897 | 163 | — | — | 0·473 | 79 |
| Denbigh & Flint | 0·541 | 111 | 0·333 | 64 | 0·465 | 110 | 0·383 | 63 |
| Anglesey | 0·389 | 80 | 0·75 | 139 | — | — | 0·316 | 53 |
| Cheshire (survey area) | 0·317 | 65 | — | — | — | — | 0·421 | 68 |
| | | | | *Cancer of Lung* | | | | |
| Caernarvon & Merioneth | 0·109 | 23 | 0·615 | 113 | — | — | 0·322 | 56 |
| Denbigh & Flint | 0·200 | 43 | 0·444 | 78 | 0·465 | 82 | 0·399 | 67 |
| Anglesey | 0·278 | 60 | 0·50 | 95 | — | — | 0·303 | 55 |
| Cheshire (survey area) | 0·254 | 52 | — | — | — | — | 0·572 | 91 |

In order to make valid comparisons between the occupational groups in a county area the rates in Table III need to be further adjusted for differences in age distribution of men in those groups. This has been done by applying age-specific death rates from stomach cancer in all males of England and Wales to the age distributions of the occupations at the census and dividing the death rate at 20 and over in all males by the resulting calculated rate in the occupation to obtain comparability factors. The age distributions of farm, quarry and coal workers were obtained from the national census populations (Registrar General, 1957) and for the residue of occupations they were derived from the whole county populations by ages after deducting the proportions in the special groups as estimated from populations at ages 20–64 provided by the Registrar General. The rates in Table III were then multiplied by the appropriate comparability factors, giving the rates in Table IV which are age-adjusted in respect of both area and occupation. The same has been done for cancer of the lung and bronchus. The standard errors can be taken as the rate divided by $\sqrt{d}$.

TABLE IV.—*Estimated Death Rates of Men aged 20 and Over according to their past occupations, adjusted for Areal and Occupational Age differences*

| | Rates per 100,000 | | | | Difference from " Other " | | |
|---|---|---|---|---|---|---|---|
| | Farm | Quarry | Coal | Other | Farm | Quarry | Coal |
| | | | | | *Cancer of Stomach* | | |
| Caernarvon & Merioneth . | 143 | 158 | — | 66 . | $+77\pm20$ | $+92\pm26$ | — |
| Denbigh & Flint . . | 86 | 62 | 106 | 61 . | $+27\pm10$ | $-1$ | $+45\pm13$ |
| Anglesey . . . | 62 | 134 | — | 46 . | $+16\pm12$ | $+88\pm67$ | — |
| Cheshire* . . . | 50 | — | — | 65 . | $-15\pm7$ | — | — |
| | | | | | *Cancer of Lung* | | |
| Caernarvon & Merioneth . | 27 | 117 | — | 49 . | $-22\pm5$ | $+68\pm19$ | — |
| Denbigh & Flint . . | 51 | 81 | 85 | 67 . | $-16\pm7$ | $+14\pm19$ | $+18\pm11$ |
| Anglesey . . . | 71 | 94 | — | 52 . | $+19\pm13$ | $+42\pm47$ | — |
| Cheshire* . . . | 61 | — | — | 93 . | $-32\pm9$ | — | — |

* Part of county in Liverpool Hospital Region excluding county boroughs.

Comparing the occupations, the rate differences between the special groups and the residual group are shown in the table with standard errors of the differences. These indicate *excess* with probabilities of 700 to 1 or more for stomach cancer in farm workers of Caernarvon, Merioneth, Denbigh and Flint, quarry workers of Caernarvon and Merioneth and coal miners of Denbigh and Flint, and for lung cancer in the quarry workers of Caernarvon and Merioneth. On the other hand they indicate *deficiency* above the conventional significance level for lung cancer in farm workers of Caernarvon, Merioneth, Denbigh, Flint and Cheshire. It is evident from this that men who had worked habitually with soil or coal, slate or igneous rock showed pronounced excess of stomach cancer rates compared with men who had not done so, the excess being highly significant for the farm and quarry groups in the Caernarvon-Merioneth area and for farm and coal mine groups in the Denbigh-Flint area, but absent in the limestone and sandstone quarry workers of the latter area. In Cheshire however, where incidence of the disease is normal, farm workers showed no excess over other occupations. Lung cancer rates on the other hand were lower amongst farm workers, the deficiency compared with other occupations being present and significant in all areas except Anglesey ; but the quarry and mining groups showed somewhat higher rates than the residual group with a significant excess in the Caernarvon-Merioneth area where igneous rock and slate is quarried.

If the incidence of stomach cancer had been at the rates normal for rural areas and not affected by occupation the deaths of men in Caernarvon and Merioneth counties in the 3 years would have numbered 17 in the farm group, 12 in the quarry group and 72 in other occupations, instead of 50, 35 and 117 as in Table I, indicating that about 55 per cent of the total excess was accounted for by the farm and quarry workers who comprised no more than a quarter of the men at risk. This suggests that the effect of soil on stomach cancer mortality of men in this area arises to an important extent through actual contact with the earth, and if that is so it would be expected that stomach cancer mortality of the wives of farm workers would not show any considerable difference from that of wives of other workers. This can be tested in respect of the wives of men in social subclass IV*a*, agricultural workers, since during the survey in 1952–55 deaths of married women from cancer were coded according to the husband's social class, and in Table V the cancer site frequencies are shown for the subclass IV*a*, the rest of classes III–V, and classes I–II. It is estimated that one eighth of the last of these groups were wives of farmers (codes 010, 011, 020) who were not distinguished from the rest of class II in the coding ; but the Table shows that stomach cancer formed a rather smaller proportion of total cancer both in groups IV*a* and I–II than in the large residual group consisting of the rest of III–V, and it must be concluded that wives of the whole farming population showed no excess in the proportion of stomach cancer compared with other married women. It follows from the ratios to all cancer except lung and stomach by the method used for men that the estimated crude death rates from stomach cancer in the 3 groups of Table V were about 44, 39 and 40 per 100,000 at ages 20 and over, the corresponding national rates being 31, 24 and 22, so the women's excess of this form of cancer in North Wales is evidently no more pronounced on farms than elsewhere.

TABLE V.—*Cancer Deaths in Wives of Agricultural Workers (I Va) and Other Social Groups of Married Women in North Wales.*

| | Deaths in 3 years | | | Per cent of all cancer | | |
|---|---|---|---|---|---|---|
| Primary site | IVa | Rest of III–V | I–II | IVa | Rest of III–V | I–II |
| Lung and bronchus | — | 30 | 12 | — | 3·8 | 3·7 |
| Stomach | 12 | 172 | 62 | 20·3 | 21·5 | 18·7 |
| Oesophagus | 5 | 27 | 8 | 8·5 | 3·4 | 2·4 |
| Intestine and rectum | 13 | 162 | 65 | 22·0 | 20·2 | 19·6 |
| Other cancer | 29 | 409 | 184 | 49·2 | 51·1 | 55·6 |
| Total | 59 | 800 | 331 | 100 | 100 | 100 |

*History of residence with garden*

In the course of the 1952–55 survey questions were asked from relatives after deaths from cancer of certain sites, mainly the lung, stomach, intestine, rectum (males) and breast, as to the kinds of house in which the deceased person had lived during the last 20 years, and these could be classified by the code into house with a vegetable garden (denoted here as *VG*), house with a garden but not providing vegetables (denoted as *OG*) and place of residence with no garden (denoted as *NO*). A question was asked also as to whether green vegetables had been consumed habitually from such a garden. The numbers for which no answer could be obtained were small and have been excluded in calculating percentages in the three groups. In respect of lung, larynx, oesophagus, stomach, breast and rectum cancers the questions were asked throughout 3 years of the survey, whereas for intestine they were asked over a shorter period, so absolute figures are not comparable between the site groups, but since each group consists of consecutive and unselected deaths the percentages with garden are comparable. In north-west Wales, which consists of Anglesey, Caernarvon and Merioneth, the records for men assigned to the VG category about 90 per cent of farm workers, 50 per cent of quarry workers and 40 per cent of others ; and amongst all women the proportion was 42 per cent in this area, 46 per cent in Denbigh and Flint and 35 per cent in Cheshire.

TABLE VI.—*Deaths from Cancer amongst Men who had not worked in Farm, Quarry or Mine, and amongst Women, analysed by history of Residence with a Vegetable or Other Garden*

| | | | Males | | | Females | | | | |
|---|---|---|---|---|---|---|---|---|---|---|
| | | | Lung | Stomach | Other cancer | Lung | Stomach | Intestine | Breast | Other cancer |
| | | | *Possession of a garden during last 5 years* | | | | | | | |
| *Wales N.W.:* | Numbers with or without | *VG* | 25 | 54 | 72 | 9 | 58 | 52 | 62 | 42 |
| | | *OG* | 25 | 17 | 37 | 6 | 35 | 26 | 27 | 17 |
| | | *NO* | 40 | 46 | 66 | 14 | 58 | 44 | 44 | 33 |
| | Percent | *VG* | 27·8 | 46·2 | 41·1 | 31·0 | 38·4 | 42·6 | 46·7 | 45·7 |
| | | *VG + OG* | 55·6 | 60·7 | 62·3 | 51·7 | 61·6 | 63·9 | 66·9 | 64·1 |
| *Wales N.E.:* | Numbers with or without | *VG* | 51 | 48 | 111 | 8 | 72 | 49 | 56 | 41 |
| | | *OG* | 38 | 38 | 48 | 7 | 42 | 29 | 43 | 25 |
| | | *NO* | 35 | 32 | 47 | 7 | 40 | 16 | 35 | 23 |
| | Percent | *VG* | 44·7 | 40·7 | 53·9 | 36·4 | 46·8 | 52·1 | 41·8 | 46·1 |
| | | *VG + OG* | 71·8 | 72·9 | 77·2 | 68·2 | 74·0 | 83·0 | 73·9 | 74·2 |
| *Cheshire:* | Numbers with or without | *VG* | 38 | 34 | 47 | 11 | 33 | 21 | 41 | 20 |
| | | *OG* | 75 | 37 | 74 | 14 | 37 | 28 | 48 | 36 |
| | | *NO* | 40 | 29 | 40 | 5 | 20 | 12 | 24 | 14 |
| | Percent | *VG* | 24·8 | 34·0 | 29·2 | 36·7 | 36·7 | 34·4 | 36·3 | 28·6 |
| | | *VG + OG* | 73·9 | 71·0 | 75·2 | 83·3 | 77·8 | 80·3 | 78·8 | 80·0 |
| | | | *Possession of a garden during 15 years before that* | | | | | | | |
| *Wales N.W.:* | Numbers with or without | *VG* | 20 | 46 | 64 | 13 | 59 | 52 | 59 | 41 |
| | | *OG* | 26 | 20 | 32 | 6 | 34 | 25 | 26 | 14 |
| | | *NO* | 40 | 51 | 73 | 13 | 56 | 41 | 42 | 34 |
| | Percent | *VG* | 23·3 | 39·3 | 37·9 | 40·6 | 39·6 | 44·1 | 46·5 | 46·1 |
| | | *VG + OG* | 53·5 | 56·4 | 56·8 | 59·4 | 62·4 | 65·3 | 66·9 | 61·8 |
| *Wales N.E.:* | Numbers with or without | *VG* | 52 | 45 | 97 | 8 | 78 | 49 | 53 | 42 |
| | | *OG* | 37 | 36 | 48 | 4 | 37 | 23 | 39 | 25 |
| | | *NO* | 33 | 33 | 57 | 6 | 37 | 22 | 39 | 21 |
| | Percent | *VG* | 42·6 | 39·5 | 48·0 | 44·4 | 51·3 | 52·1 | 40·5 | 47·7 |
| | | *VG + OG* | 73·0 | 71·1 | 71·8 | 66·7 | 75·7 | 76·6 | 70·2 | 76·1 |
| *Cheshire:* | Numbers with or without | *VG* | 34 | 31 | 39 | 12 | 32 | 19 | 40 | 20 |
| | | *OG* | 73 | 34 | 69 | 11 | 34 | 31 | 41 | 35 |
| | | *NO* | 41 | 33 | 50 | 5 | 21 | 11 | 30 | 13 |
| | Percent | *VG* | 23·0 | 31·6 | 24·7 | 42·9 | 36·8 | 31·1 | 36·0 | 30·3 |
| | | *VG + OG* | 72·3 | 66·3 | 68·4 | 82·1 | 75·9 | 82·0 | 73·0 | 80·3 |

Table VI shows the numbers of deaths in categories *VG*, *OG* and *NO* and compares the proportions having had a vegetable garden (*VG*) and any garden (*VG + OG*) amongst men who had not worked in farm, quarry or mine, and amongst all women, according to the primary site of cancer causing death. In Wales N.W., the area of highest stomach cancer mortality, there are no significant differences between the frequencies of vegetable or any garden for stomach, intestine, breast and other cancer except lung. Men who died of lung cancer showed peculiarly low proportions with a vegetable garden compared with the stomach and other groups, the *VG* percentages during the last 5 years before onset of the disease being 27·8 ± 4·7 compared with 46·2 ± 4·6 and 41·1 ± 3·7, and during 15 years before that 23·3 ± 4·7 compared with 39·3 ± 4·5 and 37·9 ± 3·7 ; but the proportions having had a garden of any kind showed only slight deficiences. Women who died of lung cancer also showed a lower *VG* proportion than the other groups during the last 5 years but owing to the small number of lung cancers the differences are below significance level. In Wales N.E. comprising Denbigh and Flint counties there are no significant differences between the cancer groups except that men with stomach cancer showed a low *VG* proportion during the last 5 years, 40·7 ± 4·5 compared with 44·7 for lung and 53·9 ± 3·5 for other cancer, but this was not true for women. In the Cheshire area covered by this study, on the other hand, the VG were rather higher for stomach than for other cancer in men but were the same for stomach as for breast in women.

TABLE VII.—*Proportions of Decedents as in Table VI reported to have consumed Vegetables from Own Garden in last* 10 *years*

| Group | Wales N.W. | | Wales N.E. | | Cheshire | |
|---|---|---|---|---|---|---|
| | Number of deaths | Per cent affirmative | Number of deaths | Per cent affirmative | Number of deaths | Per cent affirmative |
| *Males:* | | | | | | |
| Lung | 73 | 23·7 ± 4·4 | 124 | 32·3 ± 4·2 | 154 | 20·1 ± 3·2 |
| Stomach | 121 | 34·3 ± 4·1 | 119 | 36·1 ± 4·4 | 98 | 28·6 ± 4·6 |
| Other | 162 | 30·9 ± 3·6 | 194 | 44·8 ± 3·6 | 158 | 30·4 ± 3·6 |
| *Females:* | | | | | | |
| Lung | 29 | 37·9 ± 9·0 | 19 | 21·2 ± 9·3 | 30 | 30·0 ± 8·4 |
| Stomach | 152 | 29·6 ± 3·7 | 157 | 42·7 ± 3·9 | 86 | 26·8 ± 4·8 |
| Intestine | 124 | 30·6 ± 4·1 | 96 | 41·7 ± 5·2 | 57 | 24·6 ± 5·7 |
| Breast | 132 | 33·3 ± 4·1 | 132 | 38·6 ± 4·2 | 114 | 30·7 ± 4·3 |
| Other | 91 | 39·6 ± 5·1 | 92 | 43·5 ± 5·2 | 70 | 24·3 ± 5·1 |

Table VII shows the frequencies of definite answers to the question whether green vegetables had been consumed habitually from the garden during the last 10 years, and the proportions of affirmative replies are similar to but rather lower than the *VG* proportions in Table VI. In each area men with lung cancer show lower proportions than those with stomach or other cancer, the differences from the latter group being statistically significant in two areas, but the women do not show this. There are no consistent differences between stomach and intestine, breast or other cancers.

It would appear from these results that, whatever is the reason for the connection between stomach cancer incidence in houses and the quality of the soil around them it does not arise to any important extent from the eating of green vegetables grown in the garden, nor is the incidence greater in houses having a garden than in other dwellings provided that farmers and quarry workers are first excluded.

The low proportions of lung cancer decedents who had lived in houses with a vegetable garden might be due to larger numbers of them having lived in large towns before residing in North Wales. From samples of hospital patients living in North Wales who were questioned about previous residence it appeared that 22 per cent of those with lung cancer had lived in large towns compared with 18 per cent of those with other kinds of cancer (Stocks, 1958, Table XIII), a difference which seems hardly adequate to account for the deficiencies in Tables VI and VII. Response to a question as to source of milk supply also showed that out of 215 men in North Wales who died of lung cancer and had not worked in farm, quarry or mine 36 per cent were said to have been supplied with milk from local farms, compared with 50 per cent of 228 who died of stomach cancer and 50 per cent of 338 who died of other cancer.

### SUMMARY

In the north-western counties of Wales where mortality from cancer of the stomach is very high, men who had worked at any time during 20 years before death on farms or in quarries suffered much higher death rates from this cause in a three year period than those who had not been so employed, and more than half of the total excess was accounted for by these groups In Denbighshire and Flint where stomach cancer mortality is not quite so high men who had worked on farms or in coal mines showed a similar excess in comparison with other men. In western Cheshire excluding the county boroughs, where stomach cancer mortality is normal, no such difference appeared for men who had worked on farms. Wives of agricultural workers in North Wales also showed no appreciable excess in death rates compared with other married women.

Histories of having lived in a house with a vegetable garden and of having consumed green vegetables from it were not associated with any excess of mortality from stomach cancer in comparison with other cancers excluding the lung, either amongst men who had not worked on a farm or in a quarry or mines or amongst women generally. These findings suggest that the association between excessive stomach cancer incidence and certain soil characters which has been found in North Wales does not arise through the agency of green vegetables grown on the soil, and in the case of men actual contact with the earth in their daily work would seem to be a factor of importance.

Lung cancer mortality was notably lower amongst men who had worked on farms and amongst others who had lived in houses with a vegetable garden, but was enhanced in those who had quarried slate or igneous rock.

### REFERENCES

Davies, R. I. and Wynne Griffifth, G.—(1954) *Brit. J. Cancer*, **8**, 56.

Registrar-General—(1938) *Decennial Supplement. Occupational Mortality.*—(1957) *Ibid.*

Stocks, P.—(1958) *Rep. Brit. Emp. Cancer Campgn*, **35**, Supplement.

*Idem* and Davies, R. I.—(1960) *Brit. J. Cancer*, **14**, 8.